# Experimental Investigation on the Strengthening of Reinforced Concrete Beams Using Externally Bonded and Near-Surface Mounted Natural Fibre Reinforced Polymer Composites—A Review

**DOI:** 10.3390/ma15175848

**Published:** 2022-08-25

**Authors:** John Uduak Effiong, Anthony Nkem Ede

**Affiliations:** Department of Civil Engineering, Covenant University, Ota 112233, Nigeria

**Keywords:** natural fibres, reinforced concrete, failure mechanisms, strengthening, sustainable concrete infrastructure

## Abstract

Developing more resilient and sustainable physical infrastructure increases the demand for sustainable materials and strengthening approaches. Many investigations into strengthening RC beam structures have used either externally bonded (EB) or near-surface mounted (NSM) systems with synthetic fibre reinforced polymer composites. These synthetic fibres are unsustainable since they involve the use of nonrenewable resources and a large amount of energy. Research shows that natural fibre reinforced polymer (NFRP) composites may be an alternative to synthetic FRP composites in the strengthening of concrete beams. However, there is limited literature that validates their performance in various structural applications. Hence, the purpose of this paper is to explore the advances, prospects, and gaps of using EB/NSM NFRP techniques in strengthening concrete beams to provide areas for future research directions. The NSM FRP technique provides improved strengthening effects and mitigates the concerns associated with the EB system, based on a wider range of applications using synthetic FRPs. However, the NSM NFRP strengthening technique has been underutilized, though the EB NFRP system has been more commonly explored in reviewed studies. The knowledge gaps and areas for proposed future research directions are essential in developing work in emerging NFRPs and strengthening techniques for sustainable infrastructure.

## 1. Introduction

**Figure 1:**
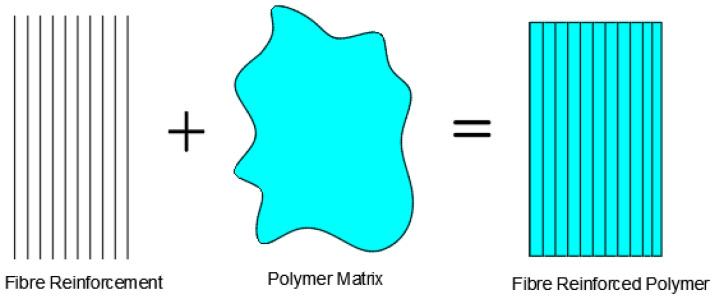
Composition of FRP composites [16].

Concrete today is a very essential component in most construction projects. Its utilization globally is seen to be immense. However, reinforced concrete structures face various levels of deterioration during and after their service life due to various forms of loading conditions and other external agencies. This explains the need for structural enhancement of deteriorating reinforced concrete infrastructure. Several studies have been conducted to evaluate the capacity of natural fibres in improving the flexural, tensile, shear, impact load, and torsional capacity of concrete in order to reduce the demand for synthetic fibres and achieve a more eco-friendly and sustainable construction [1,2,3,4,5,6]. Repairs and upgrades to the building and civil infrastructure in various regions of the globe have become a serious requirement over time. Retrofitting, at present, is used for structural enhancement of the capacity of structural components or the whole structure. The goal of retrofitting a structure is to make it more stable to meet contemporary building regulations. These include steel sheet bonding, polymer coating around concrete with the use of innovative materials in the form of FRPs, Ferro cement, and so on. FRP is a makeup of fibre reinforcements plus the polymer matrix as seen in Figure 1. The structural and mechanical characterization of the reinforcement in FRP composites dictates the mechanical properties of the composite, which in turn impacts the strength and rigidity of the composite bearing 70–90% of load [7,8,9,10,11,12]. The FRP matrix component is responsible for receiving and transferring stresses placed on the material; provides rigidity and form; protects the reinforcing fibres from chemical attack; isolates the fibres so that they can act independently; reduces the rate of the formation of the cracks; and protects the reinforcing fibres from impact and mechanical damage [13,14,15]. Thermosets and thermoplastics are two types of polymer matrices. There are two types of thermosetting materials: resins that are created by the chemical interaction of resin and hardener to generate a permanently hard resin and resins that are solid when cured but soft when heated. These thermoplastics include nylon, polypropylene (PP), polyethylene of low density or high density, and polyester resins [16].

**Figure 2:**
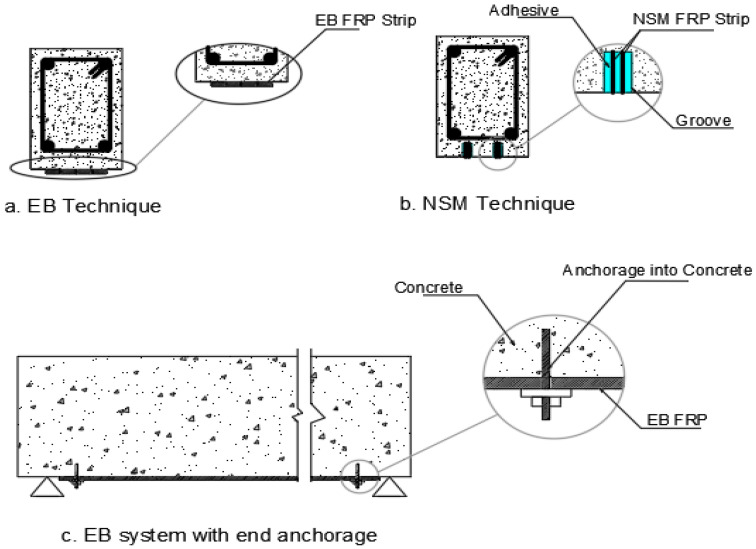
Flexural strengthening systems.

**Figure 3:**
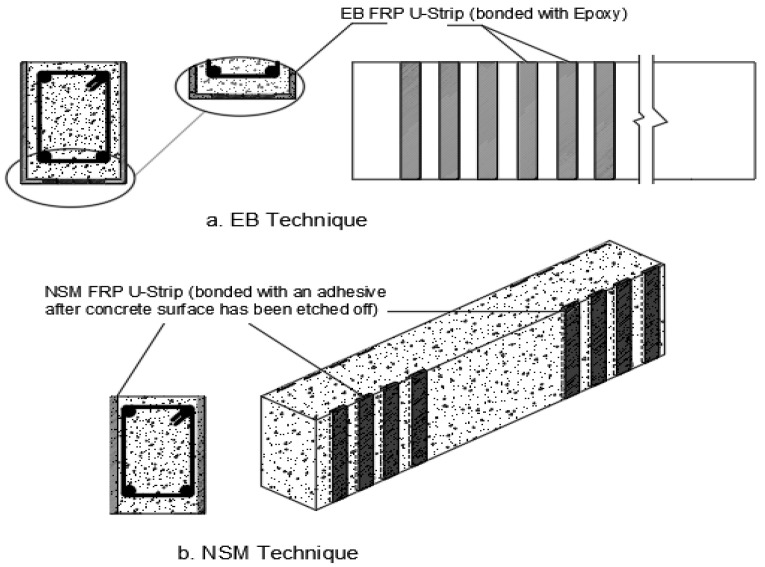
Shear and torsional strengthening systems.

**Figure 4:**
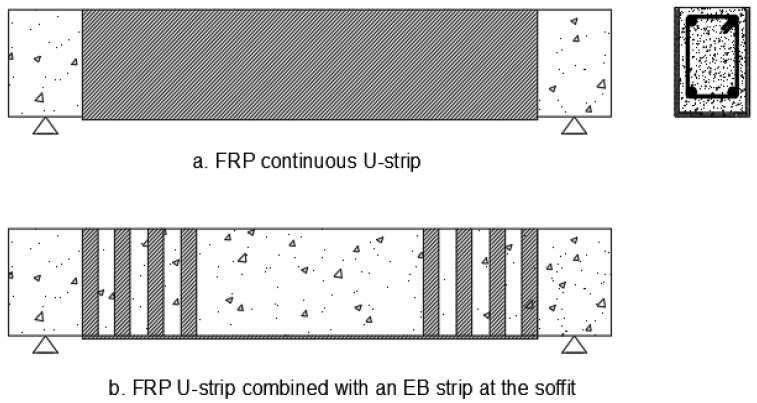
Hybrid Strengthening systems.

Fibres have been engineered over the years to form exterior bonded and near-surface mounted natural fibre composites in reinforced concrete beams, as seen in Figure 2, Figure 3 and Figure 4 for various strengthening systems. These strengthening systems include flexural strengthening, shear strengthening, torsional strengthening, and hybrid strengthening, which provide strengthening for combined structural behaviours. Studies have shown externally bonded FRPs can be applied to the soffits of RC beams using a plane FRP fabrics which are bonded in layers by the aid of an adhesive [17,18]. These are usually adopted to raise the flexural capacity of the structural beams. Also, these fabrics can be utilized to envelop the beam in the form of a U-shape, engulfing the soffit and 2 sides of the beams with the same anchoring system through the use of an adhesive. This method is most often adopted to improve the shear and torsional strength of RC beams [19,20]. It can be applied to tunneling, bridge decks, slabs, dock loading, thin unbonded overlays, concrete plinths and platforms, and concrete sheets [21]. Designing externally bonded FRP has been made easier thanks to the work of several international organizations. This guidance relies on a defined pull-out strain between FRP and reinforced concrete when utilized for bond-critical applications to examine the raise in member structural strength (e.g., ACI 440.2R-17) [22]. However, as per the studies, de-bonding occurs at low FRP strain from the concrete surface, despite the EB FRP technique’s widespread popularity. Hence, the full potential of FRP cannot be developed with this approach [23,24]. Reinforced Concrete structures such as slabs or beams may fail in a variety of ways, including concrete crushing, yielding the steel, rupturing the FRP, and failing to join at the concrete-FRP interface [25]. These can occur when concrete is subjected to a wide variety of environmental variables depending on the structure type, locale, and other variables. Thermal conditions, moisture, chemical assaults, UV radiation, salt, deicing agents, freezing and thawing cycles, and alkaline water intrusion all fall within this category [22]. Due to the noted drawback of the EB FRP system (delamination failure), NSM FRP technology has been used. As illustrated in Figure 2, the NSM FRP system begins with grooves etched into the surface of concrete and then attaches the FRP composites to these grooves with a suitable bonding material [24,26]. It was invented between 1940 and 1949 in Europe by inserting steel rods through slits cut into the surface of concrete structures using cement mortar as the idea of the NSM FRP technique [24,27]. When comparing the two FRP systems, the NSM FRP system exhibits a greater bond advantage. The NSM technique can further offer protection to the FRP composite against damage and the impacts of environmental agencies [24,28].

Several studies have explored the advancements in strengthening concrete structures using FRPs. However, these studies have focused mainly on synthetic fibres such as carbon, glass, basalt, and aramid FRPs, which are the most often utilized reinforcing materials in FRPs due to their high strength and stiffness [24,26,29,30,31]. However, these materials pose ecological concerns; hence, it has become imperative to investigate the possibility of natural fibres as an alternative. Natural fibres are less expensive than synthetic fibres such as carbon fibres, which improves their economic and scientific potential [16,32,33]. This review is aimed at exploring the advancement and assessing the efficacy of externally bonded and near-surface mounted systems using Natural Fibre Composites to strengthen RC structural beams to identify structural applications, bond behaviour, failure mechanisms, durability, gaps, and possible enhancements to provide areas for future research directions in strengthening concrete beam structures.

## 2. Natural Fibres

**Figure 5:**
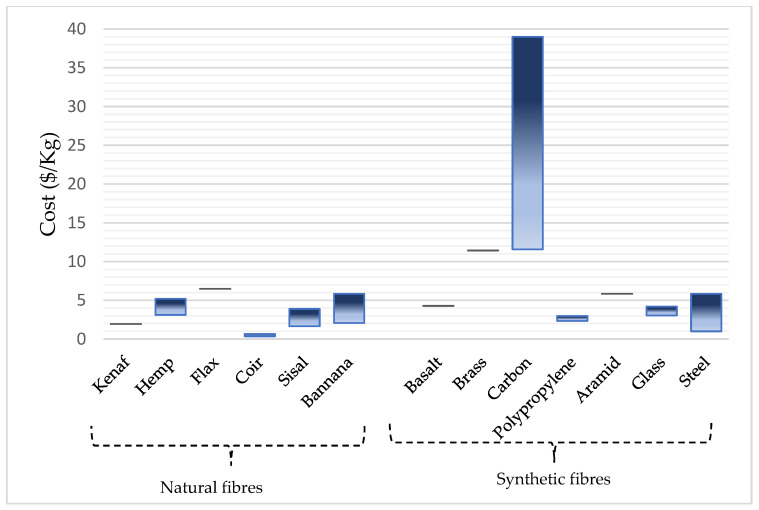
Cost comparison between natural and synthetic fibres.

**Figure 6:**
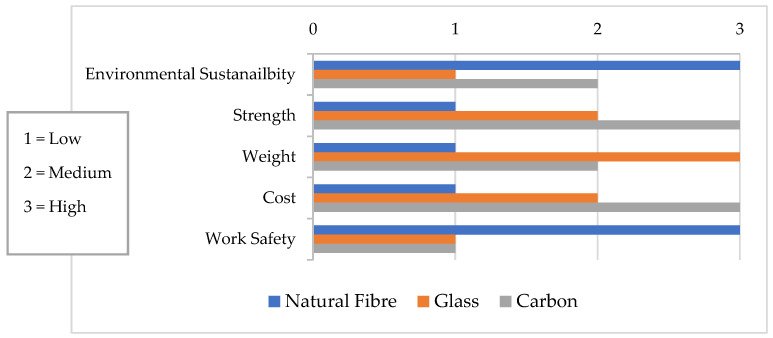
Economic, strength, and environmental comparison between natural and synthetic fibres [35,36].

An increasing number of people throughout the globe are turning to synthetic fibres such as glass, carbon, or aramid for reinforcement and retrofitting purposes. From considerable research carried out throughout the globe over the past 30 years, we have gained a greater knowledge of the characteristics and behavior of FRPs under various situations [33]. Thus, more FRPs will likely be used in the future. However, the use of synthetic fibres in rural regions is unlikely due to their relatively high cost and limited use. New revolutionary materials technologies in civil engineering have begun to find their way into the market to suit the needs of advanced infrastructure. Innovators in the fields of textiles, transportation, aviation, and construction may all benefit from advancements in science and technology because of the universal properties that make them applicable across several sectors [34]. Recent years have seen renewed interest in using natural FRP composites in place of conventional FRP materials such as glass fibre and carbon fibre because of the potential benefits of good strength-to-weight ratio, lower material prices, and natural fibres being sustainable materials, as portrayed in several studies. However, the current cost of natural fibres, as shown in Figure 5, almost measures up with some synthetic fibres because of their increasing demand and applicability in various sectors such as building, gardening, pulp and paper, cosmetic, food and automotive sectors, among others. Despite these recent findings, these fibres possess good mechanical and strength properties and they are eco-friendly and require low energy inputs, as shown in Table 1 and Figure 6.

It is true that natural fibres have flaws that must be overcome to compete with glass and carbon. Glass fibres outlast and outperform natural fibres in terms of durability and strength [37]. However, the use of high-strength naturally occurring fibres in the form of a laminate has been proven to be a more ecologically friendly and cost-effective alternative than the use of synthetic fibres for retrofitting and improving the structural integrity of the structure. Structural retrofits employ on-site or pre-cured natural fibre reinforced polymer (NFRP) laminates [32,38,39]. Due to their ease of handling, textiles consisting of woven natural fibres (fabrics) are frequently used in the manufacture of laminates. To begin with, preparation of the concrete surface by using a water jet at high pressure to eliminate any weak spots is done [40]. These textiles are put on in layers on the surface of the prepared concrete. A suitable quantity of resin is added prior to and following each fabric layer to ensure that the fibres are fully saturated with resin and that the laminate has no air pores. In this scenario, estimating the laminate’s strength capability is problematic since the exact amount of resin used is unknown. Pre-cured NFRP laminates are formed in a mold, which allows for a broader range of fibre forms than previous methods (single fibres, fibre ropes, and woven). Curing the laminate is carried out independently of strengthening the structural component. After applying the laminate to the structural component and allowing it to dry, it is glued together. While pre-cured laminate production is more precise than built-up laminate manufacturing on-site, the built-up laminate methodology is more versatile. It may be wrapped in a number of ways around RC beams and slab soffits, and with other reinforced concrete structural elements [32].

**Figure 7:**
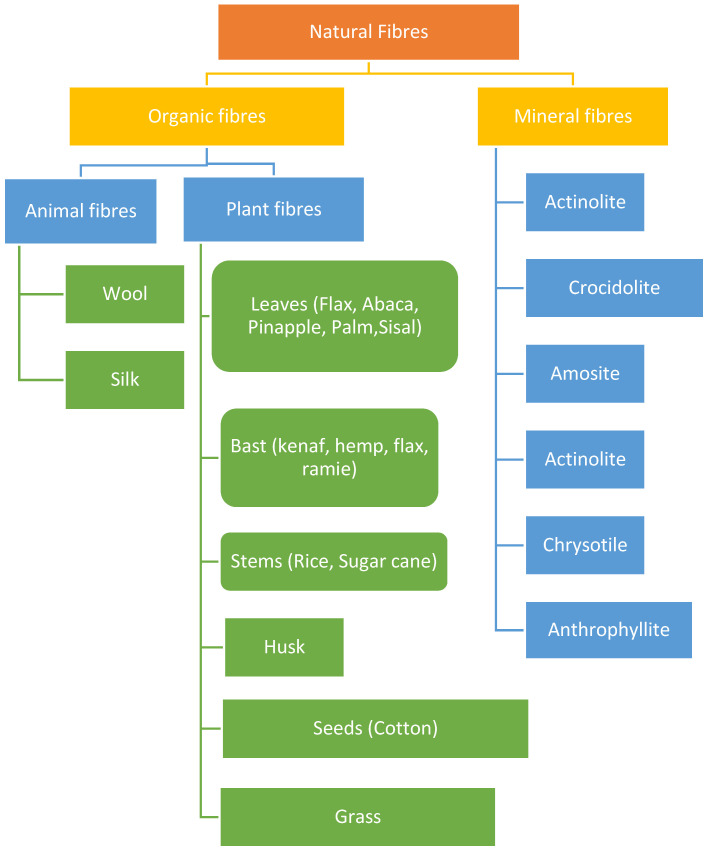
Types of Natural Fires [33].

To build a laminate that is effective at reinforcing a concrete beam, it is crucial to comprehend the characteristics, strengths, and limitations of the specific fibre functioning as reinforcement [41]. This knowledge can only be gained by studying the characteristics of natural fibre reinforced polymer laminates. These fibres may be employed in a variety of ways to make concrete stronger. As short discontinuous fibres, they can be included into fresh concrete without any additional processing, or as part of polymer laminates that are externally attached to a concrete element. Because of the multiple advantages of using natural fibres to reinforce concrete, this is a highly sought-after commodity in the building business. Because the fibres are widely accessible in nature, their extraction involves little energy and money, and they are long lasting and strong for their workability. In polymer composites, natural fibres with high strength can replace synthetic fibres as reinforcement. Natural fibres’ hydrophilic nature is a detriment to their use, although it may be remedied chemically to enhance the surface morphology of the fibres to prevent undesirable components from adhering to the fibres’ surface. Alkaline treatment (NaOH) has been demonstrated to increase fibre surface roughness by breaking hydrogen bonding. The alkaline treatment exposes short-length crystals and depolymerizes the cellulose component by removing the oils, waxes, inorganic salts, and lignin that coat the fibre’s outer surface. Excessive fibre concertation treatment can potentially reduce composite strength by causing fibre surface rupture and harming the primary and secondary walls of the fibre [36,42]. Moreover, the load bearing capacity of an RC beam is increased by more than 50% when the RC beam is strengthened using NFRP laminates rather than synthetic FRP laminates. Although the usage of NFRP composites to substitute CFRP and GFRP is a promising breakthrough in the area of reinforcing RC beams, more research into the failure mechanisms of RC beams enhanced with NFRP is required [16]. Natural Fibre Reinforced Concrete comes in a variety of forms and properties. Organic and mineral fibres are categories of natural fibres as shown in Figure 7. Leaves, bark, stems, husks, seeds, and grass are all sources of plant fibres, as are a variety of other plant parts. The skin and fur of many animals are used to make animal fibres [41,43].

Table 2 shows the mechanical properties of some natural fibres which can be used as a constituent to produce FRP composites for the strengthening of RC structures.

## 3. Research Process

**Figure 8:**
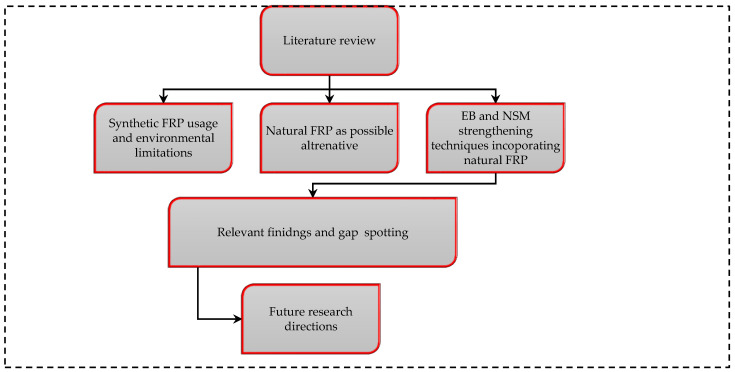
Flow chart of paper organization.

Figure 8 shows the flow chart of paper organization, which defines the review process. It is divided into six main sections, which include (i) The literature review (ii) Synthetic fibre reinforcement usage and environmental implications; (iii) Natural FRP as possible alternative to synthetic FRPs; (iv) EB and NSM strengthening techniques incorporating natural FRPs; (v) Key findings and gap spotting, and (vi) Future research directions.

## 4. External Bonded NFRP Strengthening Techniques Adopted in Past Researches

### 4.1. Flexural Applications

Use of NFRP in flexure applications is gradually gaining significant attention with the current need for a more sustainable means of strengthening deteriorating RC elements, though these studies are limited. Sen & Reddy [39] created a sisal fabric reinforced polymer composite and studied its behaviour associated with flexure and drew comparison to that of CFRP and GFRP composites. When it came to flexural strength, SFRP composites were shown to be adequate when compared to CFRP and GFRP composites. An investigation of failure mechanisms and impact on flexural behavior of RC beams wrapped in SFRP, CFRP, and GFRP, wrapped as U-wraps in one layer over the whole length of the beam, using two distinct wrapping procedures, was conducted. Researchers found that SFRC strengthening of RC beams improved flexural strength and load deflection almost equivalent to CFRP and GFRP strengthening. As shown in Table 3, the SFRC-enhanced RC beam had the best ductility and delayed crack development, whereas the CFRP-enhanced beams and the GFRP-enhanced beams failed due to FRP rupture or debonding failure, respectively, as shown in Table 3. As a natural fibre, this meant that the EB SFRP system might be employed as an alternative fabric reinforcement in FRP for the ‘efficient strengthening of RC beams’ flexural properties.

**Figure 9:**
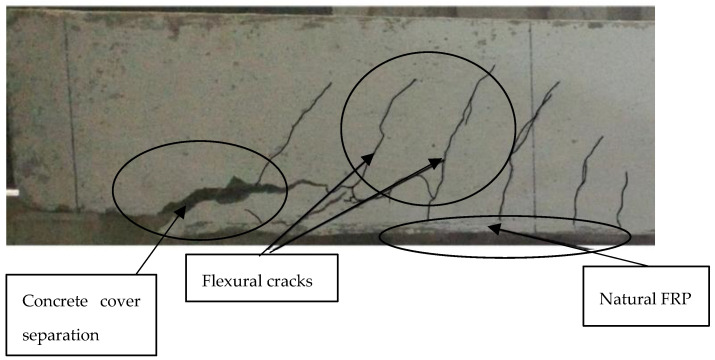
Failure mode showing concrete cover separation [16].

According to Nwankwo & Ede [32], the use of natural fibres in polymer composites may provide a more ‘sustainable alternative to synthetic fibres’. In their study, RC beams in flexure were strengthened using a KFRP laminate that was developed and produced for external bonding. For comparable loads, kenaf fibre laminates enhanced ultimate load capacity by 82% and decreased deflections in the RC beam. In comparison to the control beam, the KFRP-enhanced beam exhibited a deflection that was 43% lower at the yield load. To boost the RC beam’s ductility, KFRP-strengthened beams were used instead of the control beam. The failure mode reported in this study was failure due to concrete cover separation as shown in Figure 9. The results of the numerical analysis were all in good agreement with the findings of the empirical study. However, because of the numerical model’s ideal circumstances, the ANSYS beams were stiffer. Experimental and numerical predictions differed by around 1.15% when it came to the strength of the control beam, and by 5.36% when it came to its ultimate load. ANSYS’s fracture pattern was also in agreement with the experimental beams.

Also, for RC buildings, Grazide et al. [45] investigated the effectiveness of wood composite reinforcement (wood + FRP) in order to reduce reflection costs and utilize more environmentally friendly, but ‘poor’ construction materials instead. A single epoxy bi-component was used to assemble both the wood plus FRP and the wood plus FRP plus R C beam assemblies without needing any time between the two assemblies (wood + FRP and R C beam). The findings indicated an increase in ultimate strength capacity by 40–130%. The wide variation in findings was attributable to the predominance of knots, the primary flaws in the bending strength of natural material and other wood complexities.

**Figure 10:**
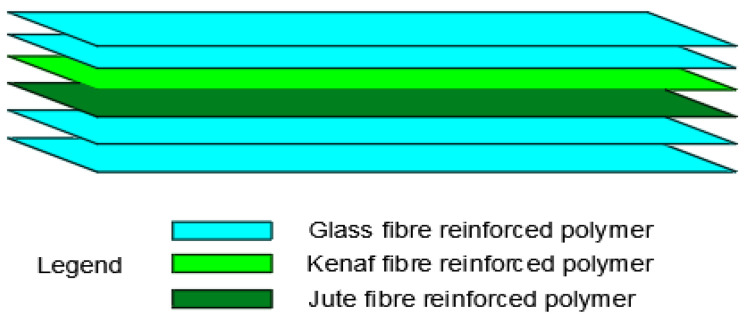
Stacking sequence of optimal hybrid FRP in flexure, incorporating natural fibres with overall thickness of 2.3 mm [46].

For enhanced strengthening, some researchers propose merging synthetic fibres with natural fibres to better the mechanical and structural strength of RC constructions because of the downsides (such as increased water absorption and lower mechanical strength qualities when compared to glass fibres) of employing natural fibres [46]. This is termed hybridization. A study by Chethan et al. [46] looked at the effects of different laminate stacking orders of E-glass/kenaf/jute fabric epoxy resin plates on the tensile and flexural behaviour of RC beams as shown in Figure 10. Increasing the amount of glass plies significantly improved the mechanical characteristics of kenaf and glass fibre hybrid laminates, compared to jute and glass fibre hybrid laminates up to 244 MPa in flexure.

**Table 3:** Comparison of RC beam flexural strengthening results using EB natural composites.

References	Beam Specimens IDs	Fibre Utilized	Area of FRP Composite (mm^2^)	Wrap Config	Longitudinal Rebar Ratio (%)	Ultimate Load	Load Type	Deflection at Midspan (mm)	Failure Mode	Strength Increase (with Comparison with Relevant Control) %
Sen & Reddy [39]	Con1, Con2				0.90	80.00	Flexure	11.43	Flexure	
	SF1, SF2	Sisal	99.50	U-Wrap at 90° (continuous)	0.90	170.00		37.58	Concrete cover cracking, formation of flexure cracks on beam, and FRP afterwards	112.50
	CF1, CF2	Carbon	18.00	U-Wrap at 90° (continuous)	0.90	200.00		16.31	Rupture of FRP and formation of flexure crack in the beam	150.00
	GF1, GF2	Glass	21.00	U-Wrap at 90° (continuous)	0.90	180.00		17.63	Debonding of FRP and formation of flexure crack in the beam	125.00
	SF3, SF4	Sisal	99.50	U-Strip wrap at 90°	0.90	130.00		26.99	flexure crack in the beam	62.50
	CF3, CF4	Carbon	18.00	U-Strip wrap at 90°	0.90	120.00		10.13		50.00
	GF3, GF4	Glass	21.00	U-Strip wrap at 90°	0.90	110.00		10.85		37.50
Nwankwo & Ede [32]	CB				0.86	53.94		12.02		-
	SB	Kenaf	1625.00	EB Strip along soffit	0.86	98.07	Flexure	8.32	Concrete cover separation	81.81
Grazide et al. [45]	REF				0.69	48.30	Flexure	12.00		
	W25_1	Wood plank	2250.00	EB laminate along soffit	0.69	71.90		7.40	failure of the wood on the tensile side	48.86
	W25_2	Wood plank	2250.00		0.69	108.00		11.40		123.60
	W45_1	Wood plank	4050.00		0.69	119.20		8.50	shear failure with concrete cover debonding	146.79
	W45_2	Wood plank	4050.00		0.69	113.20		9.00		134.37
	W45_CFRP9_1	Wood plank + CFRP rod (9 mm)	4050.00		0.69	111.20		8.50	composite debonding from the end of the element	130.23
	W45_CFRP9_2	Wood plank + CFRP rod (9 mm)	4050.00		0.69	122.50		8.30		153.62
	W45_GFRP9_1	Wood plank + GFRP rod (9 mm)	4050.00		0.69	117.70		6.80		143.69
	W45_GFRP14_1	Wood plank + GFRP rod (14 mm)	4050.00		0.69	118.00		7.00		144.31
	W45_GFRP14_2	Wood plank + GFRP rod (14 mm)	4050.00		0.69	114.50		6.20		137.06
Joyklad et al. [38]	1-CON				2.14	22.60	Flexure	125.00	flexure	
	2-A-1L	Jute	220.00	EB Strip along soffit	2.14	28.00		94.50	flexure	23.89
	3-A-3L	Basalt	220.00	EB Strip along soffit	2.14	29.00		118.00	flexure with rupture of the FRP	28.32
	4-B-1L	Jute	300.00	U-Strip wrap at 90°	2.14	31.00		141.50	flexural cracks with greater deflections	37.17
	5-B-3L	Basalt	300.00	U-Strip wrap at 90°	2.14	33.00		82.00	flexural cracks with rupture of the FRP	46.02
Yinh et al. [47]	Control				1.31	479.00	Flexure	31.68	Flexure	-
	P-2-L	Sisal	720.00	EB Strip along soffit (No anchorage)	1.31	545.00		11.30	Debonding	13.78
	P-2-L-AN	Sisal	720.00	EB Strip along soffit (with anchorage)	1.31	616.00		19.10	Intermediate crack	28.60
	P-4-L-AN	Sisal	1440.00	EB Strip along soffit (with anchorage)	1.31	650.00		20.80	Intermediate crack	35.70
	E-2 L	Sisal	720.00	EB Strip along soffit (No anchorage)	1.31	571.00		12.53	Debonding	19.21
	E-2 -AN	Sisal	720.00	EB Strip along soffit (with anchorage)	1.31	692.00		19.71	Intermediate crack	44.47
	E-4 L-AN	Sisal	1440.00	EB Strip along soffit (with anchorage)	1.31	804.00		17.31	Intermediate crack	67.85
Ignacio et al. [48]	Control Beam				0.11	26.66	Flexure	75.89		
	1-Layer CFRP Beam	Carbon	115.82	EB Strip along soffit (No anchorage)	0.11	58.69		34.93	Critical diagonal crack debonding failure	120.14
	2-Layer CFRP Beam	Carbon	231.65	EB Strip along soffit (No anchorage)	0.11	54.49		-	Critical diagonal crack debonding failure	104.39
	10-Layer GFRP Beam	Hemp	965.20	EB Strip along soffit (No anchorage)	0.11	44.72		40.40	Horizontal rupture failure of GNFRP	67.74

N/B: CFRP—Carbon fibre reinforced polymer; GFRP—Glass fibre reinforced polymer.

In research by Joyklad et al. [38], jute fibre reinforced polymer composites (JFRP) and basalt fibre reinforced polymer (BFRP) composites were used to strengthen small-scale RC beams. The findings show that RC beams’ ultimate load-bearing capability may be greatly improved by including both JFRP- and BFRP-reinforcing materials. Natural sisal fibre reinforced polymer composites were shown to be more effective than conventional strengthening techniques in increasing the strength and flexibility of reinforced concrete elements. Because they are made from natural materials, natural sisal FRPs are environmentally friendly and sustainable.

Considering a variety of polymer matrices, the FRP thickness and compressive strength of concrete, Yinh et al. [47] discovered that using natural sisal FRPs is considerably beneficial in improving the strength and ductility of constrained concrete in the experiments. Increasing sisal FRP thickness results in an improvement in load-carrying capability and ductility. However, its effectiveness in providing external confinement and improving strength has been found to be lower for high-strength concrete than for low-strength concrete. This agrees with a study conducted by Ignacio et al. [48] using hemp fibres in comparison to CFRP. The study proved that despite the fact that CFRP showed better strengthening capacity, there was an additional cost to increasing stiffness in the CFRP-strengthened beam; the failure mode went from being flexible to brittle. According to Pingulkar et al. [49], increased fibre content in laminated composites has been shown to also improve energy absorption and impact resistance. It is worthy to note that compressive strength of concrete had little influence on how well-enhanced specimens perform, which is similar to study carried out by Hawileh et al. [50]. However, RC beams reinforced with natural sisal FRP are susceptible to failure due to FRP debonding from the concrete surface. To mitigate this, a novel anchoring mechanism was devised and tested to see whether it could prevent the FRP from detaching from the concrete surface. Due to its superior mechanical qualities, epoxy resin was chosen over polyester resin in this application. Based on trial findings, this anchoring mechanism was determined to be successful in keeping natural sisal FRPs attached to the concrete surface [47].

It is worthy to note that corrosion is a significant contributor to the degradation of concrete [51,52]. However, little study has been done on the impacts of corrosion on NFRP reinforced structures. Research has been conducted primarily employing synthetic FRPs. According to an experimental investigation by Yang et al. [51], FRP laminates with U-wrappings, placed immediately without replacing the degraded concrete cover, were shown to be a viable option for strengthening corroded beams. The strengthening system was efficient in raising the load-carrying capacity and flexural stiffness despite average corrosion levels of 20%, local corrosion levels of up to 57%, and corrosion-induced cracks of up to 1.9 mm wide by 70% and 135% for GFRP and CFRP, respectively (Yang et al., 2021). Hu et al. [53], on the other hand, removed and replaced damaged concrete covers with hybrid-engineered cementitious composite (ECC) with CFRP fabrics affixed to the soffit of the ECC layer. Corrosion ratios lower than 10% were shown to be optimal for ECC strengthening, restoring the beam’s capacity to that of a virgin one. Due to ECC’s numerous micro-cracking characteristics, the flexural capacity of a beam was greatly increased by the hybrid CFRP-ECC strengthening. ECC’s remarkable ability to control cracking prevented the remaining concrete of higher strength from failing.

### 4.2. Shear Applications

Jute fibre reinforced polymer (JFRP) composites were investigated by Jirawattanasomkul et al. [19] for usage as an exterior strengthening fabric for reinforced concrete (RC) beams in the region of shear. JFRP greatly enhanced the shear strength of beams, per the findings of the tests and as shown in Table 4. It was shown that when properly repaired and reinforced with appropriate layers of JFRP, shear-deteriorated RC beams may completely recover their previous shear strength. The pre-damaged beams encased with JFRP had a greater shear strength than the initial shear strength, which was tested before damage. They also suggested that to improve the structural performance of damaged beams when using JFRP for RC, it is also necessary to use the right strengthening ratio and repair techniques. The failure mode of all reinforced beams was a rupture of the JFRP sheets in their study.

Alam et al. [52] developed a theoretical model for shear-strengthened KFRP laminated beams. Shear and KFRP laminate rupture caused failure of the KFRP-strengthened beams, whereas the CFRP-strengthened beams failed due to flexural shear and subsequent delamination of the laminate. RC beams were reinforced with CFRP and KFRP laminates KFRP and CFFR laminates both improved the strength of beams in a quite similar manner, although KFRP had a shear fracture load of 100% more than that of the control beam. The control beam’s ultimate load capacity was increased by 32.85% with KFRP and by 34.31% with CFRP. Delamination in CFRP laminates occurred before they could reach their full capacity; this did not occur with KFRP laminates.

EB Beams with shear-inadequate apertures have yet to be strengthened with NFRP composites through this approach. However, strengthening has been applied using synthetic FRPs as shown by Aksoylu et al. [54], who conducted an experiment in which two CFRP composites were employed to strengthen the shear deficient beams with circular holes. Findings showed that the strengthening approach was satisfactory though factors such as the hole size, locations, and geometry of the apertures contribute to the degree of strengthening.

### 4.3. Blast Resistance

**Figure 11:**
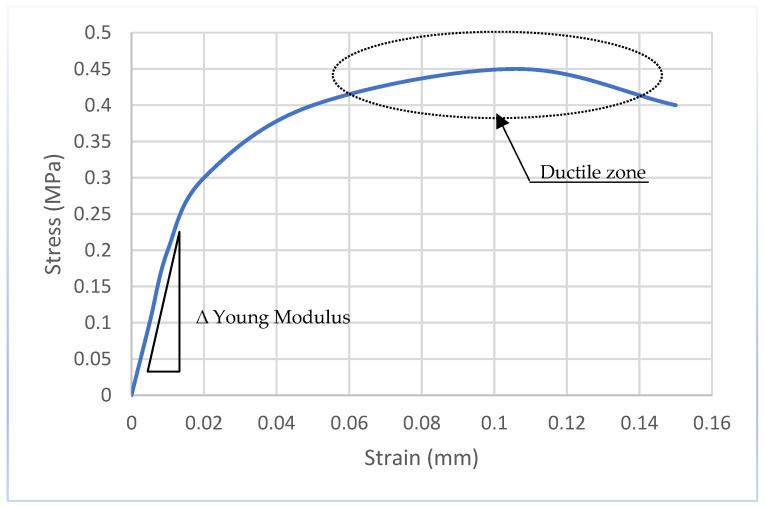
Typical stress-strain diagram of hemp reinforced concrete [57].

The demand for safer buildings is becoming more important as the frequency of purposeful and inadvertent explosions continues to rise globally [55]. Draganić et al. [55] conducted a study that reviewed and systematized the studies on blast load reduction. Experimental studies were used in the design and retrofit methodology investigations. The findings of field testing are roughly representative of real-life scenarios. The study looked at a broad range of materials that are utilized in the design and refit of structural parts, from fibre-reinforced concrete to concrete that has been externally retrofitted. All the tested approaches demonstrated a great potential for strengthening blast resistance, as well as minimizing or even preventing flying debris. Direct comparison and performance assessment are problematic because of considerable variability in blast loading conditions and structural qualities, particularly when considering the absence of scientific agreement on standardized blast load measurements. Field tests provide important information on the behaviour of structural parts that have been enhanced in ways to withstand blast loads. Due to considerable variability in the parameters of the performed tests, a general conclusion on the efficacy of a specific material utilized for design or retrofit augmentation of structural components could not be clearly ascertained. To create comparable findings and draw conclusions for the optimum structural protection, research should be conducted in such a manner that blast load test settings are standardized. The key to providing a platform for comparison and analysis of test findings is to define standardized blast tests. The use of natural materials in the design and retrofit process might be the next feasible research topic. Hemp has been shown to have several structural health benefits [55]. Hemp fibres have a high tensile strength and ductility, as seen in Figure 11, making them ideal for FRC. Hemp fibres can be utilized as fibre reinforcement both in fresh concrete mix and as a retrofitting technique. Study results have shown that hemp fibres can be used to undertake low-cost and minimally invasive seismic interventions without disrupting the use of a structure, while at the same time providing adequate strength and ductility as C-FRP materials [56].

### 4.4. Torsion Applications

Even though torsional stresses can be induced in several structural concrete buildings, design engineers mostly ignored torsion prior to the 1960s. It was anticipated that torsional impacts were small and that the substantial safety factors utilized in flexural design would take care of them. Many incidents of torsional strain and failure have resulted from this notion [58]. The application of NFRP to torsion resistance remains unexplored [20]. Hence, research is suggested in this direction to enhance eco-friendly construction and rehabilitation work to enhance RC structures subjected to torque effects. Hence, research is suggested in this direction to enhance eco-friendly construction and rehabilitation work to enhance RC structures subjected to torque effects. However, notable research has been conducted using glass [59], carbon [60], and aramid fibre reinforced polymers [61], and this research has shown a considerable increase in cracking and ultimate strength and ultimate twist effect of torque for strengthened concrete beams enhanced with these synthetic fibres.

## 5. Near-Surface Mounted FRP Strengthening Techniques Adopted in Past Research

### 5.1. Flexural Applications

The NSM fibre reinforced polymers (FRP) approach is gaining traction as a viable substitute to externally bonded reinforcement (EBR) due to its enhancing of the load bearing capability of RC members. When compared to the EBR approach, the NSM FRP process offers various benefits, including a lower chance of debonding and more durability [62]. However, the applicability of this innovative system has not been thoroughly investigated or compared to the EB system using NFRP composites. It is critical to conduct research in this approach as we work toward eco-friendly RC concrete advancements and advances. To provide a foundation for this research requirement, studies involving the use of synthetic FRP are reviewed.

Khalifa [62] compared the efficacy of NSM and EBR approaches for enhancement of RC beams in flexure. In his observation, it was observed that strengthening RC beams using the NSM approach enhanced the beams flexural capacity by 12–18%. On the other hand, Triantafyllou et al. [63] investigated the behaviour reinforced concrete beams with corroded steel rebars that had a low mass loss (approximately 7.5%) but required the taking out of the concrete cover with fissures, treatment of rebars, patching with cement mortar, and application of externally bonded EBR and NSM FRP composites. Thus, it showed that the near surface mount approach increased the ultimate bearing of the corroded beams by 43%, whereas NSM offered ultimate load capacity by 69% and corrosion-related cracks were determined to be wider than 0.3 mm.

Fire occurrences can also be a source of structural deterioration which could be managed using the NSM FRP strengthening technique. This was shown in research conducted by Jadooe et al. [64], which involved analysis of reinforced concrete beams of similar geometric dimensions, strengthening (using NSM CFRP composites), and loading and support configurations. The study showed that the NSM approach could improve the ultimate load capacity and stiffness of the beams exposed to high temperatures (600–700 °C), though less than the stiffness offered by the original beams not exposed to high-level temperatures.

In finding an alternative polymer matrix other than polyester resin or epoxy resin, Al-Saadi et al. [40] investigated the efficiency of the NSM system using CFRP rods with a high-strength, self-consolidating cement-based adhesive (with graphene as constituent) to improve beam flexural capacity under fatigue loading. Using the cement-based adhesive (IHSSC-CA) instead of epoxy glue improved the serviceability of NSM CFRP-enhanced and restored reinforced concrete element, per the results of the study. Similar findings were found in a study by Al-Mahmoud et al. [65], though it involved the use of CFRP rods in place of CFPRP strips. However, Zhang et al. [66] discovered NSM CFRP strips outperform other sectional forms of NSM FRP rods and bars due to the former’s smaller sectional area-to-perimeter ratio. This was examined in a review to evaluate the efficiency of near surface mounted CFRP rods or bars to CFRP sheets or laminates. This implies a larger sectional area of the NSM FRP plays a good role in enhancing better flexural capacity of an RC beam. On the other hand, Chen et al. [67], demonstrated that through an analytical model which compared with an experimental one, increasing the bond length and compressive strength of concrete improves the flexure capacity of the beam under fatigue loading. This is inconsistent with studies [49,50] carried out using EB FRP systems that indicated that an increase in compressive strength of concrete does not improve the flexural capacity of RC beams.

In other ways to improve the efficiency the NSM FRP system in strengthening RC beams, the side near surface mounted system was investigated by Abdallah et al. [68] through an experimental program. The study showed that the SNSM technique using CFRP rods was more effective in improving the load bearing capacity of the RC beam specimens. However, ductility was reduced at peak load. However, The SNSM strengthening technique was proposed as a substitute for the NSM method, and in certain situations, it may be utilized to avoid non-traditional failure modes caused by deterioration of the NSM enhancement system, such as the CFRP rod pulling out or premature debonding failure.

Many studies have been undertaken on single span beam elements, but relatively few on continuous span beams. However, Abdallah et al. [69] compared the utilization of NSM and side-NSM-FRP rods to raise the flexural strength capacity of double-span continuous concrete beams. The investigation showed both the NSM and side NSM systems had almost the same effect on strengthening the beam configuration in flexure (18–64%). Strengthened beam on the hogging and sagging zone showed better flexural capacity. However, this might not be very practical in most cases in building structures where a wall or RC column stands as an obstruction in the hogging zone.

### 5.2. Resistance to Dynamic Loadings

RC beams with pinned and free ends were studied by Capozucca [70], who examined the behaviour of both undeteriorated and deteriorated RC beam structures. Two RC beams were put through their grooves using NSM glass fibre reinforced polymer-enhanced testing (GFRP). In the compressive zone, GFRP strips were utilized to structurally enhance one of the beams. The RC beams were damaged by concrete cracking during bending testing on one of the beams and by fabricated notches on the other beam. When tested to failure bending loads, the NSM GFRP rod strengthening approach showed sufficient results, with no loss of concrete cover adhesion. Compression was followed by bending up to collapse, and debonding buckles developed only when the compression zone of concrete got crushed. The experimental dynamic testing showed that the theoretical and actual frequencies for the first two modes of the unstrengthened and undamaged beams with end pin bonds were quite close, with a maximum deviation of 10%. In addition, the study shows beams deteriorated with concrete cracking and those reinforced with GFRP rod/strip show that strengthening restricts the deteriorated state of the concrete with minimal frequency changes, even under large moments in bending. This was in correlation with a similar study conducted by Capozucca & Magagnini [71] utilizing CFRP lamina. They suggested that using non-destructive vibration testing would help engineers determine the extent of beam damage using hinged ends while simultaneously recording changes in frequency values and an increase in damage. Controlling the availability of CFRP-laminated beams may be done using this way [71]. Numerical simulations employing the finite element approach were used to predict the reaction to the free vibration of specimens (FEM), which correlated with the outcomes of the experimental procedures.

### 5.3. Bond Behaviour

Minimal research has been documented on the bonding behavior of NSM FRP composites and concrete adopting epoxy glue and evaluated under monotonic loading conditions. Seracino et al. [23] discovered that when CFRPs with high aspect ratios (width/thickness) are used, the effectiveness of the bonding system increases. Cruz & Barros [72] observed that when CFRPs with a long-bonded length are used, the pull-out capacity increases. Based on the experimental findings, Teng & fei Chen [30] computed the local relations of bond strength slip. Pullout capacity may be enhanced by ensuring the bonded length of CFRP and the NSM’s size of groove, are increased according to Novidis et al. [73]. This is in correspondence to newer study conducted by Chen et al. [67]. Galati & De Lorenzis [74] determined that bond strength may be enhanced by using a harder epoxy glue and increasing the groove size of the NSM. According to Al-Saadi et al. [40], the NSM FRP technique exhibited a better cohesion to the EB FRP technique. Additionally, he stated that a cement-based adhesive (IHSSC-CA) might be a viable alternative to epoxy glue, since it increases the serviceability of NSM FRP structurally enhanced and patched RC elements. This is contrary to studies by Al-Mahmoud et al. [65], who stated the reverse, though the cement-based adhesive adopted was different. Soliman et al. [75] discovered that after 200 °C, the freezing and thawing cycles and the pull-out capability of an epoxy glue decreased by 8% to 14% in comparison the reference samples, but it decreased by 30% to 45% for a cement-based adhesive polymer. Yun et al. [76] postulated that there is a gradual decrement in bond strength from the start to the end of the test for NSM GFRP beam samples subjected to high fatigue load conditions. However, there is only a slight decrement in bond strength at the start of the experiment for NSM GFRP beam samples subjected to low scale fatigue loading. According to Seracino et al. [23], in push–pull test findings, they developed a numerical approach to estimate the ultimate load for the debonding of intermediate cracks on beam structures. Experimental and predicted failure loads had a strong correlation, according to this study. Sena Cruz et al. [77] discovered the local bond-slip relation in reference to experimental debonding data utilizing analytical and numerical methods. FE was also utilized in the model reaction of the examined samples, taking into consideration the influence of epoxy glue on the global behavior. It discovered a high degree of correlation between FE analysis and experimental outcomes. Galati & De Lorenzis [74] developed a model through analytic studies to forecast the local bond stress-slip relationship and gauged it using data from NSM direct experimental shear testing with utilizing FRP bond length of a small degree. When used in conjunction with additional experimental variables, this model could replicate NSM FRP samples’ behavior. Analysis and experimental data for lengthy FRP-bonded length specimens show a high correlation.

**Figure 12:**
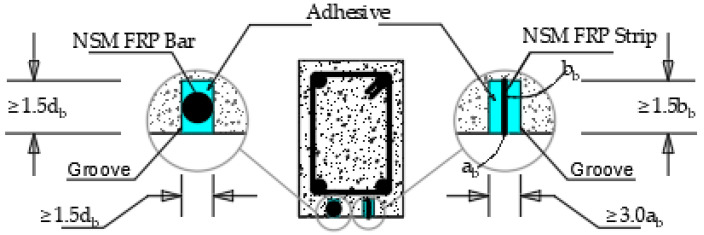
Groove Dimensions for NSM FRP Strengthening systems [78].

The cohesion failure in a small layer of concrete at the epoxy interface is usually the desired debonding failure mechanism for an NSM FRP-to-concrete contact. If the concrete and CFRP surfaces are correctly prepared and a suitable adhesive is used, then this failure scenario is conceivable to encounter [66]. NSM CFRP strips to concrete interfaces have been the subject of several local bond slip and bond strength models. For single NSM FRP strip-to-concrete connections, several of them can now provide precise forecasts for the proper length of the concrete edge. Thus far, debonding failures in NSM FRPs RC beams have been identified in four distinct ways through extensive experimental research; compressive concrete crushing failure, rupture failure, intermediate crack-induced debonding failure, and end debonding failure are all examples of the failure mechanism associated with the NSM system. RC beams made of NSM CFRP are more susceptible to concrete cover separation than interfacial debonding. Al-saadi et al. [24] discussed the factors impacting the flexural property of enhanced RC beam structures using the NSM system in their review work. From the study, it was concluded that parameters such as groove dimensions, NSM fibre reinforcement polymer clear width and edge length, bonded length, polymer matrix, and limits of tensile stress in steel reinforcement affect the NSM strengthening technique. Minimum groove dimensions for NSM FRP strengthening systems based on ACI 440.2R-08 (2008) are shown in Figure 12. d_b_ is the bar diameter and a_b_ and b_a_ are the smallest and largest widths of the FRP strips shown, respectively.

### 5.4. Shear Applications

There are only a few studies on the shear behavior of RC beams reinforced with the NFRP NSM system as seen on Table 5. Ferrier et al. [79] conducted an experiment to see whether natural flax-reinforced polymer might be employed as a supplementary shear reinforcement in reinforced concrete structures. The results revealed that flax fibre strengthening polymer had the same structural capability as carbon fibre in the shear reinforcement. The findings also show that the presence of transverse stirrups in the reinforcing zone lessened the positive impacts of flax fibres to increasing shearing capacity of beams reinforced using the EBR technique, which is consistent with the impact of stirrups on shear resistance. Stirrups had no effect on the FFRP contribution in the situation supported by the NSM technique.

Other investigations on the shear enhancement of RC beams using the NSM system have mostly used CFRP and GFRP as test materials. Studies such as these are important because they provide the groundwork for future research in the field of NFRP. Nonlinear finite element models and practical investigations were carried out by Banjara & Ramanjaneyulu [80] to investigate the effects of shear deficiency and GFRP reinforcement on reinforced concrete beams. Their investigation revealed that effective shear strengthening of RC beams depends on factors such as orientation of reinforcement fibres in the fabrics and adhesive utilized and tensile stresses in reinforcement. NSM CFRP applications in prestressed concrete prisms have also been done in an experimental and analytical study by Deng et al. [81] to examine the shear performance of reinforced beams structures. In comparison to the non-strengthened control beam, a 65.23% to 103.45% increase was revealed with the utilization of NSM CFRP composites. A good match was also found between the analytical findings and test results of the performance of reinforced beams structures. In comparison to the non-strengthened control beam, a 65.23% to 103.45% increase was revealed with the utilization of NSM CFRP composites. A good match was also found between the analytical findings and test results.

### 5.5. Seismic Application

**Figure 13:**
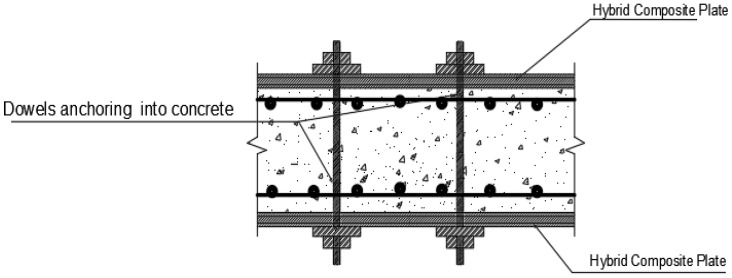
Hybrid composite plate retrofitting process with dowels anchoring into RC specimen [82].

A wide gap is also seen in the application of the NSM system using NFRP in seismic loading since there little or no studies to validate their applicability and efficiency. However, few studies using CFRP have been conducted to test the effectiveness of NSM system in seismic retrofitting. Esmaeeli & He [82] in their study revealed the efficacy of the NSM system (utilizing both a hybrid composite plate and strain hardening cementitious composite) in repairing and strengthening a seismic-damaged RC beam column joint connection. A dowel and chemical anchor system, as illustrated in Figure 13, was significant in slowing down the pull-out process at the strain hardening cementitious composite-concrete connection.

## 6. Durability Performance of Concrete Structures with NFRP

Reinforcement in polymer composites may be made more environmentally friendly by using natural fibres instead of synthetic fibres. Although natural fibres must be used in greater amounts to give the same strength as synthetic fibres, they are a considerably more cost-effective and rational solution. Reinforced concrete members using NFRP laminates were shown to be more resistant to cyclic stress and to fail at greater displacement values than CFRP laminates. NFRP laminate strengthening of concrete has a bright future thanks to the elimination of most of the objections to the external bonding technique using the near surface mounting method (NSM) [41]. However, durability performance in terms of water penetration, alternate wetting and drying, ultrasonic velocity, temperature resistance, abrasion resistance, and effect of saline environment on concrete members with NFRP is yet to be extensively explored using both strengthening systems.

## 7. Findings and Relevant Gaps in Knowledge

It has been discovered that employing natural fibre reinforced polymers (EB or NSM) to strengthen RC beams provides a variety of issues, possibilities, and recommendations, some of which are consistent with earlier studies. Table 6 shows key findings and gaps that are essential to be filled to fully embrace and utilize NFRPs in reinforcing RC structures.

## 8. Future Research Directions

On the basis of a thorough evaluation of previous research and the identification of research gaps, the following are the suggested future research areas for strengthening concrete beams in a sustainable and robust manner utilizing natural FRP.

- Studies concerning the use of NSM natural FRP in strengthening RC beams are scant. This implores the need for further research into such a technique to provide better strengthening to RC beams.
- Numerical analysis will be required for the validation of experimental findings associated with EB and NSM natural FRP beam strengthening to proffer wider acceptance and applicability.
- Further studies into the life cycle assessment of the various strengthening techniques using natural FRP will be essential to assess the environmental performance and help improve future strengthening techniques. This is relevant considering the constant changes in the socio-economic and environmental climate.
- Research into green alternatives to conventional polymer matrices such as epoxy and polyester is encouraged, as they require significant energy input in the manufacturing process and could be toxic, thereby reducing the positive environmental compatibility of natural FRPs.
- This review also suggests the adoption of innovative technology (such as smart sensors) to give improved insights into the long-term behavior of EB and NSM NFRP reinforced concrete structures.
- Application of natural FRP in improving torsional resistance of concrete beams is underexplored, hence the need for research is essential as torsional effect is one that can be experienced often in dynamic loading conditions such as strong winds and seismic loads.
- Research into the application of natural FRPs in the strengthening of beams with unconventional physical dimensions, such as beams with openings, is essential for wider applicability.
- The need to explore the effectiveness of NFRP in blast resistant beam structures is also essential owing to increasing threats and the need for safer structures as a result of the increasing number of explosions worldwide.
- There are limitations to research and related issues, particularly with respect to the effectiveness of EB and NSM natural FRP composite systems when coupled loading conditions such as fatigue, cyclic, seismic, impact, and exposure to harsh weather conditions are applied. Research incorporating coupled loading conditions would be valuable to real-life applications of EB and NSM natural FRP strengthening techniques.

## 9. Conclusions

This review focused on assessing the efficacy of EB and NSM NFRP strengthening procedures in RC beams in terms of structural efficiency empirically. Based on the reviewed studies, the following conclusions can be drawn:

- GFRP and CFRP have been widely used in structural retrofitting based on reviewed studies either using the EB or NSM system. Nonrenewable resources and considerable energy use are required to produce these synthetic fibres, making them unsustainable.
- Reviewed studies have shown that natural fibre reinforced polymer (NFRP) composites may be an alternative to conventional synthetic FRP composites in structural strengthening.
- However, their use to validate their performance in various structural applications is underexplored, particularly in their application using the NSM strengthening technique.
- The NSM strengthening technique using FRPs reduces the flaws associated with the EB technique. These flaws include: delamination, concrete cover separation, and the spread of crushing shear cracks in concrete.
- Shear and rupture of NFRP are the most frequent mechanism of failure for externally side-wrapped and u-wrapped systems, whereas rupture of the NFRP is the most common cause of failure for fully wrapped systems owing to the confinement effect.
- NSM synthetic FRPs have shown failure mechanisms through flexural failure by crushing of compressive concrete, flexural failure by rupture, intermediate crack driven pull-out, and end pull-out. However, these structural behaviours remain underexplored using natural FRPs.
- NFRP strengthening also aids in the transition of a structure from a brittle to a flexible mode of failure via NFRP strengthening.
- Factors affecting the structural improvement of RC beams employing NSM system include groove sizes and FRP dimensions; clear gaps and edge lengths of NSM FRP; FRP bond length; adhesive material type; and stress restrictions in steel reinforcements. These findings are mainly associated with the use of synthetic FRPs, which are not sustainable.
- With the development of new techniques that maximize NFRP strength, minimize brittleness and increase ductility, reduce the risk of high temperature exposures and accidental damage, minimize embodied carbon as well as carbon footprints during production, and reduce high capital costs, NFRP adoption, and utilization will grow even more.

## Figures and Tables

**Table 1:** Energy input of FRP composition [33,35].

FRP Composition		Energy Input (MJ/Kg)
Natural fibres	Sisal	2.5
	Flax	2.8
	Hemp	4.2
Synthetic Fibres	Glass	13–32
	Carbon	183–286
	Steel	30–60
	Aluminium	196–257
Polymer Matrix	Polyester	63–78
	Epoxy	76–80

**Table 2:** Mechanical properties of some natural fibres [16,44].

Fibre	Density (kg/m^3^)	Tensile Strength (N/mm^2^)	Modulus of Elasticity (GPa)	Elongation at Break (%)
Flax	1500	345–1100	27.6	2.7–3.2
Jute	1300–1450	393–773	13–26.5	1.16–1.5
Kenaf	1260–1450	295–930	53	2.7–6.9
Sisal	1500	468–640	9.4–22	3.0–7.0
Coir	1150	131–175	4.0–6.0	15–40
Hemp	1470	690	70	2.0–6.0
Bamboo	600–1100	140–230	11.0–17.0	4.0–7.0
Banana	1350	529–914	8.0–32.0	3.0–10.0
Ramie	1440–1500	400–938	61.4–128	4
Rice	1650	449	1.21–1.25	2.2
Oil palm	700–1550	248	3.2	25

**Table 4:** Comparison RC beam shear strengthening results using EB natural composites.

References	Specimens	Fibre Utilized	A_sv_/S_v_	Wrap Config	Longitudinal Rebar Ratio (%)	Ultimate Load (KN)	Load Type	Deflection at Midspan (mm)	Failure Mode	Strength Increase (with Comparison to Relevant Control)
Jirawattanasomkul et al. [19]	D1-EB2-NR	Jute	0.48	U-Wrap at 90° (continuous)	5.13	195.10	Shear	21.40	Shear, rupture of JRFP	24.00
	D1-EB2-R	Jute	0.48	U-Wrap at 90° (continuous)	5.13	173.70	Shear	18.00	Shear, rupture of JRFP	11.00
	D1-EB4-R	Jute	0.96	U-Wrap at 90° (continuous)	5.13	209.50	Shear	21.20	Shear, peeling of concrete, rupture of JRFP	33.00
	D2-NS (Control)		-		5.13	196.20	Shear	-	Shear	-
	D2-EB4-NR	Jute	0.96	U-Wrap at 90° (continuous)	5.13	155.50	Shear	14.50	Shear, rupture of JRFP	1.00
	D2-EB4-R	Jute	0.96	U-Wrap at 90° (continuous)	5.13	245.40	Shear	26.40	Shear, peeling of concrete, rupture of JRFP	56.00
Alam et al. [52]	CB				1.24	137.00	Shear	-	Shear	
	KFRP	Kenaf	5.45	EB Strip along 2 sides of beam spaced at 110 mm c/c spanning the entire beam length	1.24	182.00	Shear	-	KFRP rupture and shear	32.85
	CFRP	Carbon	1.09	EB Strip along 2 sides of beam spaced at 110 mm c/c spanning the entire beam length	1.24	184.00	Shear	-	Flexural Shear	34.31

N/B: A_sv_—Area of shear fibre strip; S_v_—Spacing of Strip.

**Table 5:** RC beam shear strengthening results of EBR and NSM with flax and carbon FRP composites [79].

References	Specimens	Fibre Utilized	A_sv_/S_v_	Wrap Config	Longitudinal Rebar Ratio (%)	Ultimate Load (KN)	Load Type	Deflection at Midspan (mm)	Failure Mode	Strength Increase (with Comparison to Relevant Control) %
Ferrier et al. [79]	R0-Ref				2.93	159.00	Shear	7.50	Shear	
	R150-Ref				2.93	173.00	Shear	6.60	Shear	8.81
	R0-EBR-cont-2FFRP	Flax	10,000.00	EB continuous U-Strip spanning 500 mm (of the beam length with no stirrups from one end of the beam support)	2.93	187.00	Shear	10.20	Shear	17.61
	R0-NSM-2x150-4FFRP	Flax	20,000.00	NSM continuous Strip along 2 sides of beam (Spanning 500 mm from one end of the beam support)	2.93	176.00	Shear	8.00	Shear	10.69
	R150-EBR-cont-2FFRP	Flax	10,000.00	EB continuous U-Strip spanning 500 mm (of the beam length with 150 mm spaced stirrups from one end of the beam support)	2.93	206.00	Shear	8.70	Shear	19.08
	R150-EBR-150-3FFRP	Flax	100.00	EB U-Strips spaced at 150 mm c/c spanning 500 mm (of the beam length with 150 mm spaced stirrups from one end of the beam support)	2.93	194.00	Shear	10.00	Shear	12.14
	R150-EBR-200-3FFRP	Flax	75.00	EB U-Strips spaced at 200 mm c/c spanning 500 mm (of the beam length with 150 mm spaced stirrups from one end of the beam support)	2.93	194.00	Shear	10.00	Shear	12.14
	R150-NSM-150-4FFRP	Flax	133.33	NSM Strip along 2 sides of beam spaced at 150 mm c/c (Spanning 500 mm from one end of the beam support)	2.93	206.00	Shear	10.00	Shear	19.08
	R150-NSM-2×150-4FFRP	Flax	133.33	NSM Strip along 2 sides of beam spaced at 150 mm c/c (Spanning 500 mm from one end of the beam support)	2.93	206.00	Shear	10.00	Shear	19.08
Ferrier et al. [79]	T0-Ref				3.89	92.50	Shear	4.40	Shear	
	T180 Ref				3.89	123.00	Shear	7.40	Shear	
	T0-EBR-cont-4FFRP	Flax	28,800.00	EB continuous U-Strip spanning 500 mm (of the beam length with no stirrups)	3.89	123.40	Shear	7.00	Shear	33.41
	T0-EBR-180-4FFRP	Flax	160.00	EB U-Strips spaced at 180 mm c/c spanning 500 mm (of the beam length with no stirrups)	3.89	104.40	Shear	5.60	Shear	12.86
	T0-EBR-180-CFRP	Carbon	40.00	EB U-Strips spaced at 180 mm c/c spanning 500 mm (of the beam length with no stirrups	3.89	97.30	Shear	6.40	Shear	5.19
	T180-EBR-cont-4FFRP	Flax	28,800.00	EB continuous U-Strip spanning 720 mm (of the beam length with no stirrups)	3.89	133.00	Shear	9.50	Shear	8.13
	T180-EBR-180-4FFRP	Flax	160.00	EB U-Strips spaced at 180 mm c/c spanning 720 mm (of the beam length with no stirrups)	3.89	132.00	Shear	10.70	Shear	5.66
	T180-EBR-180-CFRP	Carbon	40.00	EB U-Strips spaced at 180 mm c/c spanning 720 mm (of the beam length with no stirrups)	3.89	125.60	Shear	10.70	Shear	2.11

N/B: A_sv_—Area of shear fibre strip; S_v_—Spacing of Strips.

**Table 6:** Gaps in knowledge based on the literature review of past studies.

S/N	Authors	Key Findings	Gaps
1	[32,46,48,51,53]	EB FRP strengthening technique shows effectiveness in flexural strengthening RC beams in modern day retrofitting	Delamination is the most frequent mechanism of failure for EB FRP systems which eventually easily yield to other forms of failure, such as concrete cover separation, concrete crushing, and shear crack propagation
2	[24,32]	NSM strengthening system stands out as a viable alternative to the EB system owing to its higher fatigue strength, ability to reduce the possibility of debonding, and capacity to protect against external agents of deterioration	Comparisons of the two systems are underexplored using NFRP composites and this is necessary to contribute to the efforts to attain better eco-friendly enhancement of structures
3	[19,32,83]	Deterioration due to debonding, residual stress, and dependability of EB NFRP-strengthened structures can all be assessed and predicted using theoretical models	Models are underexplored empirically using the NSM NFRP strengthening system
4	[32]	NFRP strengthening aids in the transition of a structure from a brittle to a flexible mode of failure via EB NFRP strengthening	This remains underexplored using NSM NFRP system (Ductility)
5	[62,66,68]	The major failure modes for NSM system include: concrete cover separation; flexure failure by crushing of compressive concrete; flexural failure by rupture; intermediate crack induced debonding; and end debonding failure	Reported failure modes are underexplored using NSM NFRP system
6	[24]	Bonding behaviour of NSM FRP system shows a better efficiency when compared to the EB system under various kinds of loading conditions. Showed the efficiency of NSM FRP system on solid RC structures	There is limited research on the use of NSM NFRP composite systems in torsional strengthening of RC beams to validate bond behaviour. Additionally, the structural behaviour of NSM FRP composites in strengthening RC beams with openings remains grossly underexplored
7	[16,66,80]	NFRP composites show plenty of promise in enhancing structures under various forms of loadings with both the EB and NSM systems	There are limitations to research and related issues, particularly with respect to the effectiveness of EB and NSM NFRP composites systems when coupled loading conditions such as fatigue, cyclic, seismic, impact, and exposure to harsh weather conditions are applied The need to explore the effectiveness of NFRP in blast resistant structures is also essential owing to increasing threats and the need for safer structures as result of an increasing number of explosions worldwide

## Data Availability

Not applicable.
